# Supplementary material for: Heat transfer control using a thermal analogue of coherent perfect absorption
Source: Nat Commun. 2022 May 13;13:2683. doi: 10.1038/s41467-022-30023-1 (PMC9106689; doi:10.1038/s41467-022-30023-1)
Supplement: Supplementary file 1 — Supplementary Information [file 41467_2022_30023_MOESM1_ESM.pdf]

Supplementary Note 1

Here, we establish the correspondence between 1D electromagnetic (EM) wave scattering and 2D heat diffusion.

1. Governing equations

For 1D wave scattering, the electric field $E(x,t)$ satisfies

$$\frac{\partial^2 E}{\partial x^2} = \varepsilon\mu \frac{\partial^2 E}{\partial t^2} \tag{1}$$

where ε is the permittivity, μ is the permeability, x is the spatial coordinate, and t is time. We focus on the scalar field $E(x,t)$ which is the magnitude of the electric field in any certain direction.

For 2D heat diffusion, according to Eq. (2) in the main text, the temperature field $T(x,\theta)$ satisfies

$$\frac{\partial^2 T}{\partial x^2} = -\frac{\partial^2 T}{\partial \theta^2} \tag{2}$$

where $x = \ln r$. We see that Supplementary Equation (2) can be mapped to Supplementary Equation (1) as follows

$$r \to e^x, \theta \to t, -1 \to \varepsilon\mu \tag{3}$$

Under the above relations, the temperature field $T(x,t)$ can be mapped to the electric field $E(x,t)$. Moreover, for time-harmonic cases, the fields are periodic in time: $E(x,t) = \mathrm{Re}[E(x)e^{i\omega t}]$, where ω is frequency. On the other hand, the temperature field is also periodic on the angular coordinate θ: $T(x,\theta) = \mathrm{Re}[F(x)e^{im\theta}]$, where m is an integer. It is thus reasonable to regard θ as a "pseudo time" in our case, with

$$m \to \omega \tag{4}$$

2. Forms of fields

Next, we consider the 1D wave scattering process. As in Fig. 1a of the main text, a slab with permittivity ε and permeability μ is placed in the interval [x_1, x_2]. The background has permittivity ε_0 and permeability μ_0. The electric field in the slab and on both sides can be written as

$$\begin{aligned} &E(x) = Ae^{ikx} + Be^{-ikx} && x_1 \le x \le x_2 \\ &E_1(x) = A_1 e^{ik_0(x-x_1)} + B_1 e^{-ik_0(x-x_1)} && x \le x_1 \\ &E_2(x) = A_2 e^{-ik_0(x-x_2)} + B_2 e^{ik_0(x-x_2)} && x_2 \le x \end{aligned} \tag{5}$$

where $k_0 = (\varepsilon_0\mu_0)^{1/2}\omega$, $k = (\varepsilon\mu)^{1/2}\omega$. A_1 and A_2 (B_1 and B_2) are the amplitudes of the incoming (outgoing) waves.

For 2D heat diffusion, following Eq. (3) of the main text, the temperature fields are

$$\begin{aligned} &F(x) = Ae^{ikx} + Be^{-ikx}, && x_2 \le x \le x_1 \\ &F_1(x) = A_1 e^{ik_0(x-x_1)} + B_1 e^{-ik_0(x-x_1)}, && x \ge x_1 \\ &F_2(x) = A_2 e^{-ik_0(x-x_2)} + B_2 e^{ik_0(x-x_2)}, && x \le x_2 \end{aligned} \tag{6}$$

where $k_0 = k = -mi$, which is consistent with the mappings in Supplementary Equations (3) and (4). Therefore, the fields in both cases have the same form

$$F(x) \to E(x) \tag{7}$$

except that x_1 and x_2 are ordered differently.

3. Matching conditions

At the interfaces, the matching conditions for the electric fields are

$$\begin{aligned} &E_1(x_1) = E(x_1), && E_2(x_2) = E(x_2) \\ &\frac{1}{\mu_0}E_1'(x_1) = \frac{1}{\mu}E'(x_1), && \frac{1}{\mu_0}E_2'(x_2) = \frac{1}{\mu}E'(x_2) \end{aligned} \tag{8}$$

By substituting Supplementary Equation (5), the matching conditions can be summarized by the transfer matrix

$$\begin{pmatrix} B_2 \\ A_2 \end{pmatrix} = \mathbf{M}_E \begin{pmatrix} A_1 \\ B_1 \end{pmatrix} \tag{9}$$

Combining Supplementary Equations (5) and (8) gives the form of $\mathbf{M}_E$ for the electric fields as

$$\mathbf{M}_E = \begin{pmatrix} 1 & 1 \\ Y_0 & -Y_0 \end{pmatrix}^{-1} \begin{pmatrix} \cos(k\Delta x) & \frac{1}{Y} i \sin(k\Delta x) \\ Yi \sin(k\Delta x) & \cos(k\Delta x) \end{pmatrix} \begin{pmatrix} 1 & 1 \\ Y_0 & -Y_0 \end{pmatrix} \tag{10}$$

where $\Delta x = x_2 - x_1$. $Y_0 = (\varepsilon_0/\mu_0)^{1/2}$ and $Y = (\varepsilon/\mu)^{1/2}$ are the admittances of the background and the slab, respectively.

For 2D heat diffusion, the matching condition is

$$\begin{aligned} F_1(x_1) &= F(x_1), & F_2(x_2) &= F(x_2) \\ \kappa_0 F_1'(x_1) &= \kappa F'(x_1), & \kappa_0 F_2'(x_2) &= \kappa F'(x_2) \end{aligned} \tag{11}$$

The corresponding transfer matrix is

$$\mathbf{M} = \begin{pmatrix} 1 & 1 \\ \kappa_0 & -\kappa_0 \end{pmatrix}^{-1} \begin{pmatrix} \cosh m\Delta x & \frac{1}{\kappa} \sinh m\Delta x \\ \kappa \sinh m\Delta x & \cosh m\Delta x \end{pmatrix} \begin{pmatrix} 1 & 1 \\ \kappa_0 & -\kappa_0 \end{pmatrix} \tag{12}$$

where κ_0 and κ are the thermal conductivities of the background and the object, respectively. Using the definition of wavenumber k in the thermal case and comparing Supplementary Equation (12) with Supplementary Equation (10), the temperature field can be mapped to the electric field through

$$\kappa_0 \to Y_0, \kappa \to Y \tag{13}$$

Combining the above discussions, it is thus shown that the 2D heat transfer problem can be formally mapped to a 1D EM wave scattering problem, with the temperature field mapped to the electric field.

Supplementary Note 2

Our method can formally map the steady-state heat diffusion to wave scattering. However, as mentioned in the main text, the temperature field does not really propagate in the *r*-direction, so other physical meanings should be given to perfect absorption in heat diffusion. Here, we offer two possible interpretations. One is based on the exergy flux, the other on the heat flux magnitude.

First, note that we are considering the temperature difference $T - T_0$, where T_0 is the central and ambient temperature (In this Note we do not set T_0 to zero). This temperature difference is a useful source of thermal energy. To quantify it, we use the concept of exergy. It is defined as the maximum useful work a system can do by bringing it into thermodynamic equilibrium with the environment. In the heat diffusion process, the local exergy density χ gives the useful thermal energy stored at the local point. It is calculated as[42]

$$\chi = u - u_0 - T_0(s - s_0) \tag{14}$$

where u is the internal energy density, s is the entropy density; u_0 and s_0 are the internal energy density and entropy density of the local spot after it has reached thermodynamic equilibrium with the environment. Its governing equation is[43]

$$\frac{d\chi}{dt} = -\nabla \cdot \left[\left(1 - \frac{T_0}{T}\right)\mathbf{q}\right] - T_0 \mathbf{q} \cdot \nabla\left(\frac{1}{T}\right) \tag{15}$$

The second term is $-T_0$ times the local entropy generation rate which is always positive, so it contributes to the dissipation of the local exergy. The exergy flow $\boldsymbol{\chi}_f$ is defined through the first term as

$$\boldsymbol{\chi}_f = \left(1 - \frac{T_0}{T}\right)\mathbf{q} \tag{16}$$

Now return to our system at steady state. The temperature distribution outside the object is

$$T_1(x,\theta) = A_1 e^{x-x_1}\cos(\theta + \alpha_1) + B_1 e^{-x+x_1}\cos(\theta + \beta_1) + T_0 \tag{17}$$

For generality, we introduce phases α_1 and β_1 for the two fields. To simplify the calculation, we reasonably assume that $T - T_0$ are much smaller than T_0 such that

$$\boldsymbol{\chi}_f \approx \frac{T-T_0}{T_0}\mathbf{q} \tag{18}$$

The exergy flux in the radial direction is thus estimated as

$$\chi_{f1}{}^{r} = -\frac{\kappa}{T_0 r}\left[A_1{}^2 e^{2(x-x_1)}\cos^2(\theta+\alpha_1) - B_1{}^2 e^{-2(x-x_1)}\cos^2(\theta+\beta_1)\right] \tag{19}$$

The total exergy flux through a circle is thus

$$Q_1 = -\frac{2\pi\kappa}{T_0}\left[A_1{}^2 e^{2(x-x_1)} - B_1{}^2 e^{-2(x-x_1)}\right] \tag{20}$$

Clearly, the amplitudes A_1 and B_2 determine the total exergy flux in the $-r$ and r directions, respectively. Similarly, the total exergy flux through a circle in the interior of the object is

$$Q_2 = -\frac{2\pi\kappa}{T_0}\left[B_2{}^2 e^{2(x-x_2)} - A_2{}^2 e^{-2(x-x_2)}\right] \tag{21}$$

At the outer boundary of the object, the total exergy flux that enters (leaves) the object is proportional to A_1^2 (B_1^2). At the inner boundary of the object, the total exergy flux that enters (leaves) the object is proportional to A_2^2 (B_2^2). The perfect absorption condition in heat diffusion as we defined in the main text ($B_1 = B_2 = 0$) thus means that no exergy flux leaves the object. Namely, the useful thermal energy is transferred from the heat sources into the object at maximum efficiency. Also, more exergy flux enters than that leaves the object when $A_1^2 + A_2^2 > B_1^2 + B_2^2$, or when $|\det \mathbf{S}| < 1$.

Another physically relevant quantity is the heat flux magnitude (outside the object)

$$\begin{aligned} |\mathbf{q}_1| &= \kappa r^{-1}(T_x{}^2 + T_\theta{}^2)^{1/2} \\ &= \kappa r^{-1}\left[A_1{}^2 e^{2(x-x_1)} + B_1{}^2 e^{-2(x-x_1)} - 2A_1B_1\cos(2\theta+\alpha_1+\beta_1)\right]^{1/2} \end{aligned} \tag{22}$$

Therefore, the heat flux magnitude is a constant when $A_1 = 0$ or $B_1 = 0$, but oscillates with θ when both fields exist. The same conclusion also applies to the interior part of the object. The perfect absorption in heat diffusion thus also corresponds to a θ-independent heat flux magnitude.

Supplementary Note 3

In the main text, we always assume that the two incident fields are in phase. Here, we discuss the influence of a phase difference between them in terms of their θ-dependences. Note that it is impossible to define a phase for the radial dependence because the field does not propagate in that direction. We consider the CPA condition with

$$\kappa = \kappa_0 \frac{r_1 - r_2}{r_1 + r_2}, \mathbf{S} = \frac{r_1 r_2}{r_1^2 + r_2^2}\begin{pmatrix} 1 & 1 \\ 1 & 1 \end{pmatrix} \tag{23}$$

The scattering matrix has two eigenvalues $s_1 = 0$ and $s_2 = 2r_1r_2/(r_1^2 + r_2^2)$. The eigenvector corresponding to the zero eigenvalue s_1 is $(A_1,A_2)^T = (A_1,-A_1)^T$. Using them as the amplitudes of the input fields, CPA is realized as shown in Supplementary Figure 1a.

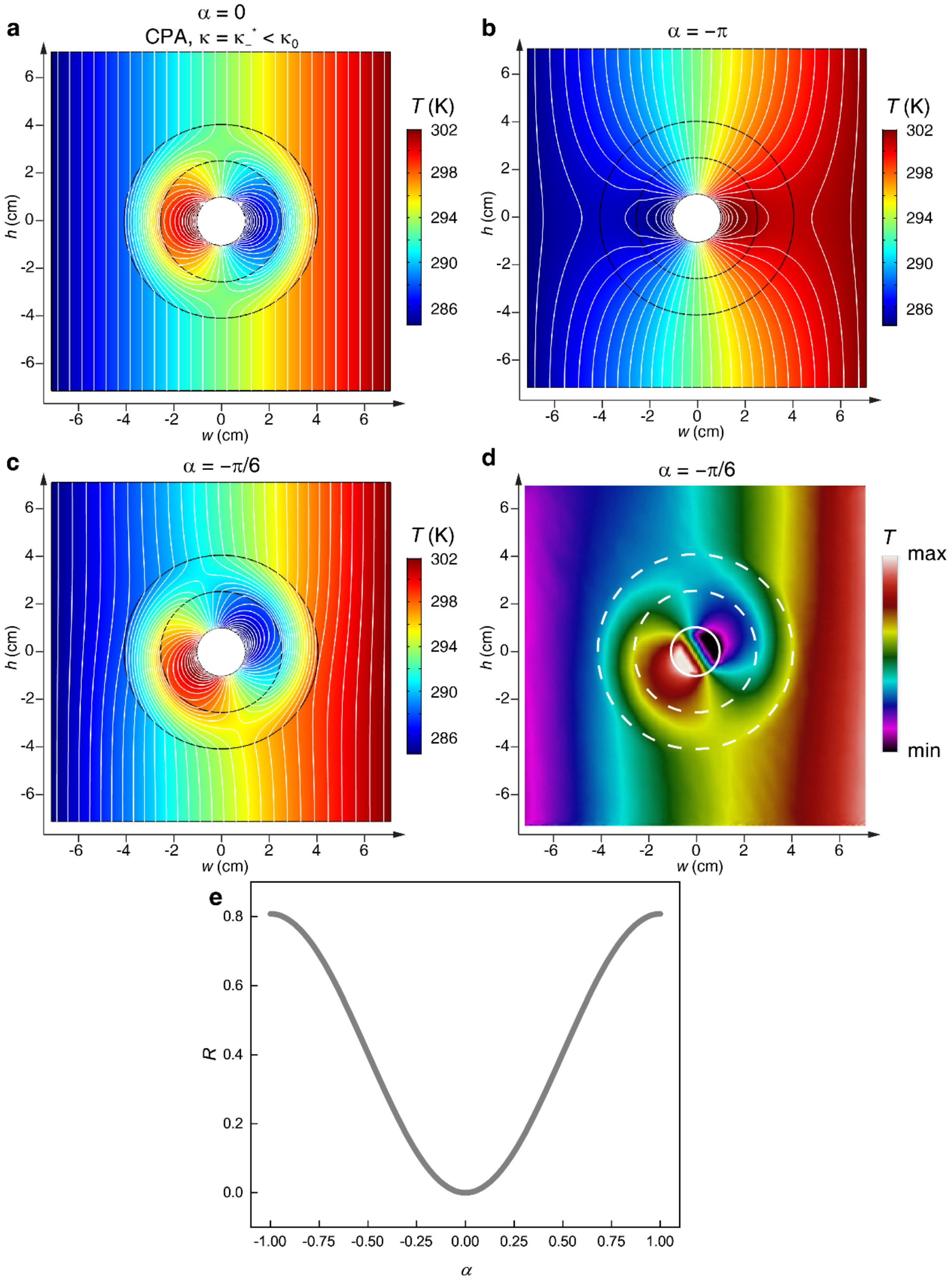

Supplementary Figure 1. Effects of a phase difference between the input fields. a, Simulated temperature distribution with unrotated input-2 field. **b**, Simulated temperature distribution with input-2 field rotated by $-\alpha = \pi$. **c**, Simulated temperature distribution with input-2 field rotated

by $-\alpha$ = π/6. **d**, Experimentally measured temperature distribution. **e**, The ratio R between exergy fluxes flowing out of and into the object.

Now we introduce a phase into A_2 such that $(A_1,A_2)^T = (A_1,-A_1e^{i\alpha})^T$. This can be easily done by rotating the input-2 field by $-\alpha$. When $\alpha = \pm\pi$, the input coincides with the eigenvector for s_2. The outgoing fields have amplitudes

$$B_1 = B_2 = A_1(1-e^{i\alpha})\frac{r_1r_2}{r_1^2+r_2^2} = A_1\sqrt{2(1-\cos\alpha)}e^{i\beta}\frac{r_1r_2}{r_1^2+r_2^2} \tag{24}$$

where $\beta = \mathrm{Arg}(1-e^{i\alpha})$. Therefore, the outgoing fields have maximized amplitudes with $\alpha = \pm\pi$. This is confirmed by Supplementary Figure 1b, where the temperature field is strongly distorted from the CPA case. It is similar to the constructive interference of the scattered fields in optical CPA, which is also achieved when the input coincides with the other eigenvector.

As shown in Supplementary Figure 1c with $\alpha = -\pi/6$, it is obvious that a small phase difference will strongly break the CPA condition and modify the temperature field. The effect is also experimentally verified by tuning the direction of the central copper bridge. The measured temperature field is shown in Supplementary Figure 1d. To quantify the influence of the phase difference, we calculate $R = (|B_1|^2 + |B_2|^2)/(|A_1|^2 + |A_2|^2)$, which gives the ratio between exergy fluxes flowing out of and into the object according to the previous discussion. The result is plotted in Supplementary Figure 1e. We can see that more exergy flux flows out of the object as α increases. At $\alpha = \pm\pi$, the input fields meet the other eigenvector of the scattering matrix, with a maximized outgoing exergy flux (around 81% of the incoming flux).

Supplementary Note 4

As another interesting example, we study the heat transfer in a background (thermal conductivity κ_0) through an elliptic object (thermal conductivity κ) and find the coherent perfection absorption

(CPA) conditions for it. As shown in Supplementary Figure 2, the outer and inner boundaries of the object are confocal ellipses with focal length $2c$ and major axes $2a_1$ and $2a_2$, respectively. Naturally, the corresponding curvilinear system is the elliptic coordinate system (ξ,η). Its relation with the cartesian coordinates (w,h) is

$$\begin{aligned} w &= c\cosh\xi\cos\eta \\ h &= c\sinh\xi\sin\eta \end{aligned} \tag{25}$$

The outer and inner boundaries of the object are then defined by $\xi = \xi_1$ and $\xi = \xi_2$.

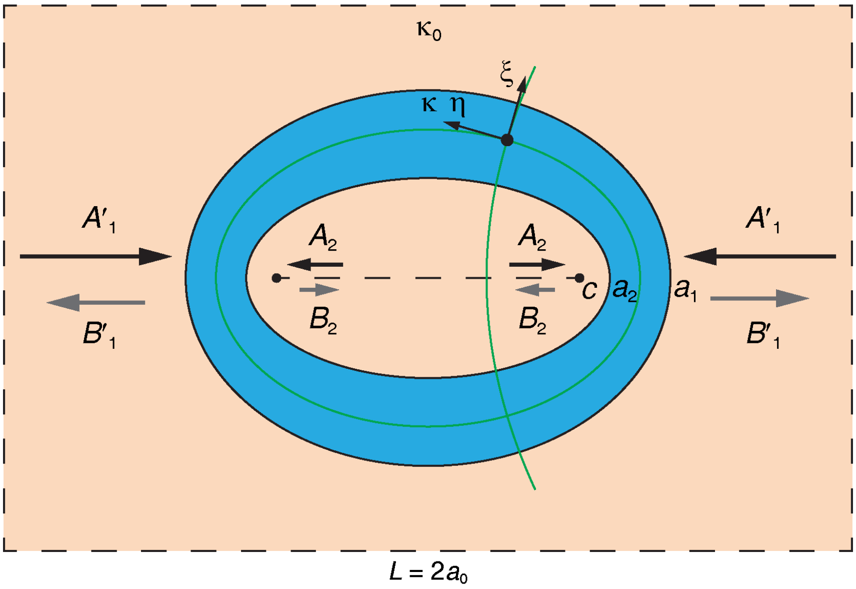

Supplementary Figure 2. Steady-state heat diffusion through an elliptic object (light blue). The black arrows represent incoming fields with amplitudes A'_1 and A_2. The grey arrows represent outgoing fields with amplitudes B'_1 and B_2. An elliptic coordinate system (ξ,η) with the same focal length $2c$ as the boundaries of the object is built (green lines).

1. Heat transfer in an elliptic coordinate system

The steady-state thermal conduction in the system follows the Laplacian equation $\nabla^2 T = 0$. In the elliptic coordinate system (ξ,η) it is written as:

$$\frac{\partial^2 T}{\partial \xi^2}+\frac{\partial^2 T}{\partial \eta^2}=0 \tag{26}$$

As in the main text, we consider "time-harmonic" solutions like $T(\xi,\eta)$ = Re[$F(\xi)e^{i\eta}$]. Following Supplementary Equation (26), $F(\xi)$ must have the form

$$\begin{aligned} F(\xi) &= Ae^{\xi}+Be^{-\xi}, & \xi_2 \le \xi \le \xi_1 \\ F_1(\xi) &= A_1 e^{\xi-\xi_1}+B_1 e^{-(\xi-\xi_1)}, & \xi_1 \le \xi \\ F_2(\xi) &= A_2 e^{-(\xi-\xi_2)}+B_2 e^{\xi-\xi_2}, & \xi \le \xi_2 \end{aligned} \tag{27}$$

The matching conditions are

$$\begin{aligned} F_1(\xi_1) &= F(\xi_1), & F_2(\xi_2) &= F(\xi_2) \\ \kappa_0 F_1'(\xi_1) &= \kappa F'(\xi_1), & \kappa_0 F_2'(\xi_2) &= \kappa F'(\xi_2) \end{aligned} \tag{28}$$

Again, we found that the problem can be mapped to the 1D wave scattering. This time it is even simpler by setting the wavenumber $k = -i$. Therefore, the transfer matrix can be directly obtained

$$\begin{pmatrix} B_2 \\ A_2 \end{pmatrix} = \mathbf{M}\begin{pmatrix} A_1 \\ B_1 \end{pmatrix} = \begin{pmatrix} 1 & 1 \\ \kappa_0 & -\kappa_0 \end{pmatrix}^{-1} \begin{pmatrix} \cosh\Delta\xi & \frac{1}{\kappa}\sinh\Delta\xi \\ \kappa\sinh\Delta\xi & \cosh\Delta\xi \end{pmatrix} \begin{pmatrix} 1 & 1 \\ \kappa_0 & -\kappa_0 \end{pmatrix} \begin{pmatrix} A_1 \\ B_1 \end{pmatrix} \tag{29}$$

where $\Delta\xi = \xi_2 - \xi_1$.

However, before we proceed, we must examine whether the input-1 A_1e^{ξ} and input-2 $A_2e^{-\xi}$ are physically meaningful. It is easy to see that input-2 is a reasonable choice since any other solution will contain nonzero e^{ξ}-component, which is a growing temperature difference away from the heat source (heat bath) and is unphysical. For input-1, we still consider the usual case of fixed boundary conditions on the left and right sides of the background. Therefore, the real input temperature field should be $T(\xi,\eta) = A_1'w = A_1'\cosh\xi\cos\eta$, such that input-1 should be $A_1'\cosh\xi$. As a result, it is more suitable to decompose the field outside the object as

$$F_1(\xi) = A_1'\frac{\cosh\xi}{\cosh\xi_1}+B_1'\frac{\sinh\xi}{\sinh\xi_1} = A_1'\cosh\xi\frac{c}{a_1}+B_1'\sinh\xi\frac{c}{b_1} \tag{30}$$

where b_1 is half the minor axis of the outer boundary of the object.

We introduce a transformation to relate it with the expression in Supplementary Equation (27)

$$\begin{pmatrix} A_1 \\ B_1 \end{pmatrix} = \begin{pmatrix} \frac{c}{2a_1} e^{\xi_1} & \frac{c}{2b_1} e^{\xi_1} \\ \frac{c}{2a_1} e^{-\xi_1} & -\frac{c}{2b_1} e^{-\xi_1} \end{pmatrix} \begin{pmatrix} A_1' \\ B_1' \end{pmatrix} \tag{31}$$

The physically relevant transfer matrix $\mathbf{M}'$ should be

$$\begin{pmatrix} B_2 \\ A_2 \end{pmatrix} = \mathbf{M}' \begin{pmatrix} A_1' \\ B_1' \end{pmatrix} = \mathbf{M} \begin{pmatrix} \frac{c}{2a_1} e^{\xi_1} & \frac{c}{2b_1} e^{\xi_1} \\ \frac{c}{2a_1} e^{-\xi_1} & -\frac{c}{2b_1} e^{-\xi_1} \end{pmatrix} \begin{pmatrix} A_1' \\ B_1' \end{pmatrix} \tag{32}$$

From which the scattering matrix $\mathbf{S}'$ is calculated as

$$\begin{pmatrix} B_1' \\ B_2 \end{pmatrix} = \mathbf{S}' \begin{pmatrix} A_1' \\ A_2 \end{pmatrix} = \frac{1}{M_{22}'} \begin{pmatrix} -M_{21}' & 1 \\ \det \mathbf{M}' & M_{12}' \end{pmatrix} \begin{pmatrix} A_1' \\ A_2 \end{pmatrix} \tag{33}$$

It is worth noting that for this hybridized scattering matrix, the parity symmetry is broken ($M'_{12} \neq -M'_{21}$), naturally because two different decompositions are used. The scattering matrix is also asymmetric ($\mathbf{S}' \neq \mathbf{S}'^{\mathrm{T}}$), since $\det \mathbf{M}' = -c^2/2a_1b_1 \neq 1$.

2. Thermal CPA for an elliptic object

The thermal CPA condition is found by solving κ from $\det \mathbf{S}' = 0$, which is explicitly written as

$$\cosh 2\gamma \cosh 2\xi_1 (\cosh 2\Delta\xi - 1) + \sinh \gamma \sinh 2\xi_1 \sinh 2\Delta\xi + 1 = 0 \tag{34}$$

where $\gamma = \ln(\kappa/\kappa_0)$. The analytical expressions of the solutions $\kappa_\pm^*$ are complicated, so it is recommended to numerically solve the equation.

In the following, we consider a concrete case where a square background with thermal conductivity κ_0 = 90 W m^{-1} K^{-1} and side length $L = 2a_0$ = 14 cm is used. For convenience, a cartesian coordinate system (w,h) is also set at the center. The focal length of the object is $2c$ = 5

cm. The major axes of its outer and inner boundaries are $2a_1 = 8$ cm and $2a_2 = 6$ cm. The input-1 is generated by applying constant boundary conditions on the left (T_1) and right (T_2) sides of the background with $A_1' = 5$ K, so $T_1 = T_0 - A_1'a_0/a_1$ and $T_2 = T_0 + A_1'a_0/a_1$, where $T_0 = 293.15$ K. A convenient property of the elliptic coordinate system is that one can generate input-2 at $\xi = 0$, which is the line segment $w = [-c, c]$ on $h = 0$. For amplitude A_2 of input-2, a linear temperature $T = T_0 + A_2w(a_2 + b_2)/c^2$ must be maintained on the segment, where b_2 is half the minor axis of the inner boundary of the object.

For the above settings, the thermal CPA condition for $\kappa > \kappa_0$ is solved as $\kappa_+^* = 4.26\kappa_0 = 383.34$ W m^{-1} K^{-1}, with $A_2 = 1.01A_1' = 5.06$ K. Numerical simulation results are plotted in Supplementary Figure 2a and e. The CPA condition for $\kappa < \kappa_0$ is solved as $\kappa_-^* = 0.18\kappa_0 = 16.50$ W m^{-1} K^{-1}, with $A_2 = -0.77A_1' = -3.86$ K. Numerical simulation results are plotted in Supplementary Figure 3b and f. We note that the results lack the simplicity and symmetry of that in a polar coordinate system (*e.g.* $\kappa_+^*/\kappa_0 = \kappa_0/\kappa_-^*$ and $A_2 = \pm A_1$). Nevertheless, the effects of CPA meet perfectly with the theoretical predictions. The temperature profiles (Supplementary Figure 3a and b) on both sides of the object are identical to the corresponding part of the input-1 (a linear profile) and input-2 (Supplementary Figure 3c and d, other parts are made translucent) fields. This is further confirmed by the temperature distributions along the cutline $h = 0$ (Supplementary Figure 3e,f).

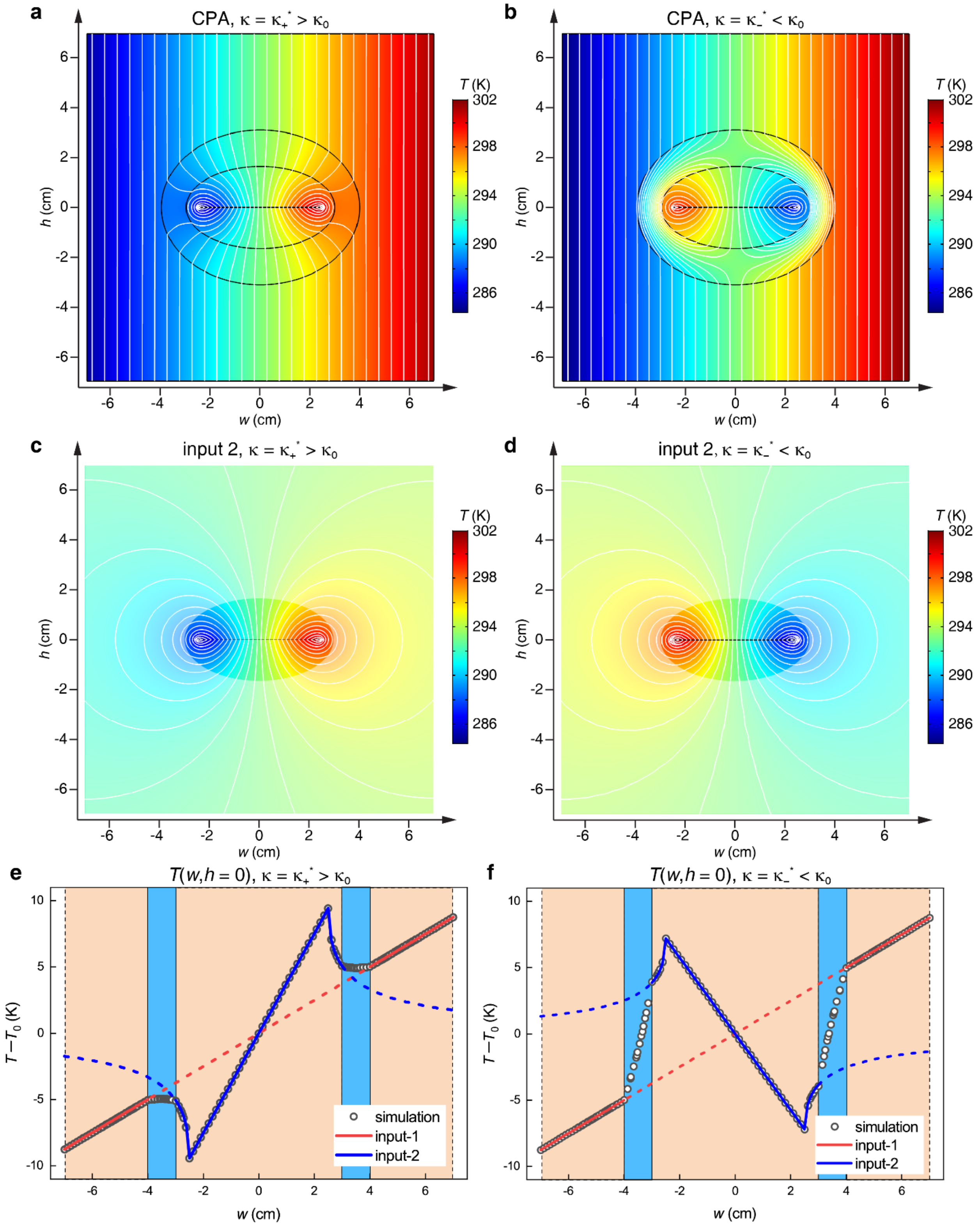

Supplementary Figure 3. Thermal coherent perfect absorption (CPA) for an elliptic object.

The thermal conductivity κ of the object is (**a**,**c**,**e**) larger or (**b**,**d**,**f**) smaller than that of the

background κ_0. **a**,**b**, The temperature distributions on the entire system with isothermal lines (white). **c**,**d**, The temperature distribution of input-2 on a pure background that is much larger (L = 1 m) than the displayed part (L = 14 cm). **e**,**f**, Temperature distributions along the line h = 0. The regions of the background (beige) and the object (light blue) are shaded. The incident fields input-1 (red) and input-2 (blue) are also plotted.

References

1. Han, T. *et al.* Theoretical realization of an ultra-efficient thermal-energy harvesting cell made of natural materials. *Energy Environ. Sci.* **6**, 3537 (2013).
2. Dede, E. M., Schmalenberg, P., Wang, C.-M., Zhou, F. & Nomura, T. Collection of low-grade waste heat for enhanced energy harvesting. *AIP Adv.* **6**, 055113 (2016).
3. Liu, W. *et al.* A Flower-Shaped Thermal Energy Harvester Made by Metamaterials. *Global Challenges* **1**, 1700017 (2017).
4. Shen, X., Li, Y., Jiang, C. & Huang, J. Temperature Trapping: Energy-Free Maintenance of Constant Temperatures as Ambient Temperature Gradients Change. *Phys. Rev. Lett.* **117**, 055501 (2016).
5. Dede, E. M., Schmalenberg, P., Nomura, T. & Ishigaki, M. Design of Anisotropic Thermal Conductivity in Multilayer Printed Circuit Boards. *IEEE Trans. Compon. Packag. Manuf. Technol.* **5**, 1763–1774 (2015).
6. Li, Y., Bai, X., Yang, T., Luo, H. & Qiu, C.-W. Structured thermal surface for radiative camouflage. *Nat. Commun.* **9**, 273 (2018).
7. Peng, Y.-G., Li, Y., Cao, P.-C., Zhu, X.-F. & Qiu, C.-W. 3D Printed Meta-Helmet for Wide-Angle Thermal Camouflages. *Adv. Funct. Mater.* **30**, 2002061 (2020).

8. Li, Y. *et al.* Transforming heat transfer with thermal metamaterials and devices. *Nat. Rev. Mater.* **6**, 488–507 (2021).

9. Yang, S., Wang, J., Dai, G., Yang, F. & Huang, J. Controlling macroscopic heat transfer with thermal metamaterials: Theory, experiment and application. *Phys. Rep.* **908**, 1–65 (2021).

10. Fan, C. Z., Gao, Y. & Huang, J. P. Shaped graded materials with an apparent negative thermal conductivity. *Appl. Phys. Lett.* **92**, 251907 (2008).

11. Narayana, S. & Sato, Y. Heat Flux Manipulation with Engineered Thermal Materials. *Phys. Rev. Lett.* **108**, 214303 (2012).

12. Guenneau, S., Amra, C. & Veynante, D. Transformation thermodynamics: cloaking and concentrating heat flux. *Opt. Express* **20**, 8207 (2012).

13. Schittny, R., Kadic, M., Guenneau, S. & Wegener, M. Experiments on Transformation Thermodynamics: Molding the Flow of Heat. *Phys. Rev. Lett.* **110**, 195901 (2013).

14. Li, Y. *et al.* Thermal meta-device in analogue of zero-index photonics. *Nat. Mater.* **18**, 48 (2019).

15. Li, Y. *et al.* Temperature-Dependent Transformation Thermotics: From Switchable Thermal Cloaks to Macroscopic Thermal Diodes. *Phys. Rev. Lett.* **115**, 195503 (2015).

16. Shen, X., Li, Y., Jiang, C., Ni, Y. & Huang, J. Thermal cloak-concentrator. *Appl. Phys. Lett.* **109**, 031907 (2016).

17. Li, Y., Shen, X., Huang, J. & Ni, Y. Temperature-dependent transformation thermotics for unsteady states: Switchable concentrator for transient heat flow. *Phys. Lett. A* **380**, 1641 (2016).

18. Li, J. *et al.* Doublet Thermal Metadevice. *Phys. Rev. Applied* **11**, 044021 (2019).

19. Li, J. *et al.* Effective medium theory for thermal scattering off rotating structures. *Opt. Express* **28**, 25894–25907 (2020).

20. Li, J. *et al.* A Continuously Tunable Solid-Like Convective Thermal Metadevice on the Reciprocal Line. *Adv. Mater.* **32**, 2003823 (2020).

21. Xu, G. et al. Tunable analog thermal material. *Nat. Commun.* **11**, 6028 (2020).

22. Zeng, L. & Song, R. Experimental observation of heat transparency. *Appl. Phys. Lett.* **104**, 201905 (2014).

23. Wang, R., Xu, L., Ji, Q. & Huang, J. A thermal theory for unifying and designing transparency, concentrating and cloaking. *J. Appl. Phys.* **123**, 115117 (2018).

24. Xu, L., Yang, S., Dai, G. & Huang, J. Transformation Omnithermotics: Simultaneous Manipulation of Three Basic Modes of Heat Transfer. *ES Energy Environ.* **7**, 65–70 (2020).

25. Han, T. *et al.* Experimental Demonstration of a Bilayer Thermal Cloak. *Phys. Rev. Lett.* **112**, 054302 (2014).

26. Xu, H., Shi, X., Gao, F., Sun, H. & Zhang, B. Ultrathin Three-Dimensional Thermal Cloak. *Phys. Rev. Lett.* **112**, 054301 (2014).

27. Li, Y. *et al.* Anti–parity-time symmetry in diffusive systems. *Science* **364**, 170–173 (2019).

28. Cao, P., Li, Y., Peng, Y., Qiu, C. & Zhu, X. High-Order Exceptional Points in Diffusive Systems: Robust APT Symmetry Against Perturbation and Phase Oscillation at APT Symmetry Breaking. *ES Energy Environ.* **7**, 48–55 (2020).

29. Xu, L. *et al.* Geometric phase, effective conductivity enhancement, and invisibility cloak in thermal convection-conduction. *Int. J. Heat Mass Transf.* **165**, 120659 (2021).

30. Qi, M. *et al.* Localized heat diffusion in topological thermal materials. arXiv:2107.05231 [physics] (2021).

31. Xu, G., Li, Y., Li, W., Fan, S. & Qiu, C.-W. Configurable Phase Transitions in a Topological Thermal Material. *Phys. Rev. Lett.* **127**, 105901 (2021).

32. Cao, P.-C. *et al.* Diffusive skin effect and topological heat funneling. *Commun. Phys.* **4**, 230 (2021).

33. Xu, G. *et al.* Diffusive topological transport in spatiotemporal thermal lattices. *Nat. Phys.* (2022) doi:10.1038/s41567-021-01493-9.

34. Kawabata, K., Shiozaki, K., Ueda, M. & Sato, M. Symmetry and Topology in Non-Hermitian Physics. *Phys. Rev. X* **9**, 041015 (2019).

35. Li, Z. et al. Non-Hermitian Electromagnetic Metasurfaces at Exceptional Points (Invited Review). *Prog. Electromagn. Res.* **171**, 1–20 (2021).

36. Yan, Q., Chen, H. & Yang, Y. Non-Hermitian Skin Effect and Delocalized Edge States in Photonic Crystals with Anomalous Parity-Time Symmetry. *Prog. Electromagn. Res.* **172**, 33–40 (2021).

37. Chong, Y. D., Ge, L., Cao, H. & Stone, A. D. Coherent Perfect Absorbers: Time-Reversed Lasers. *Phys. Rev. Lett.* **105**, 053901 (2010).

38. Wan, W. *et al.* Time-Reversed Lasing and Interferometric Control of Absorption. *Science* **331**, 889–892 (2011).

39. Baranov, D. G., Krasnok, A., Shegai, T., Alù, A. & Chong, Y. Coherent perfect absorbers: linear control of light with light. *Nat. Rev. Mater.* **2**, 17064 (2017).

40. Pichler, K. *et al.* Random anti-lasing through coherent perfect absorption in a disordered medium. *Nature* **567**, 351–355 (2019).

41. Wang, C., Sweeney, W. R., Stone, A. D. & Yang, L. Coherent perfect absorption at an exceptional point. *Science* **373**, 1261–1265 (2021).

42. Sciubba, E. & Wall, G. A brief Commented History of Exergy From the Beginnings to 2004. *Int. J. Thermodyn.* **10**, 1–26 (2007).

43. Choi, W., Ooka, R. & Shukuya, M. Exergy analysis for unsteady-state heat conduction. *Int. J. Heat Mass Transf.* **116**, 1124–1142 (2018).

44. Tan, S. & Tsang, L. Efficient Broadband Evaluations of Lattice Green's Functions via Imaginary Wavenumber Components Extractions. *Prog. Electromagn. Res.* **164**, 63–74 (2019).

45. Li, Y., Li, J., Qi, M., Qiu, C.-W. & Chen, H. Diffusive nonreciprocity and thermal diode. *Phys. Rev. B* **103**, 014307 (2021).

46. Li, J. *et al.* Reciprocity of thermal diffusion in time-modulated systems. *Nat. Commun.* **13**, 167 (2022).

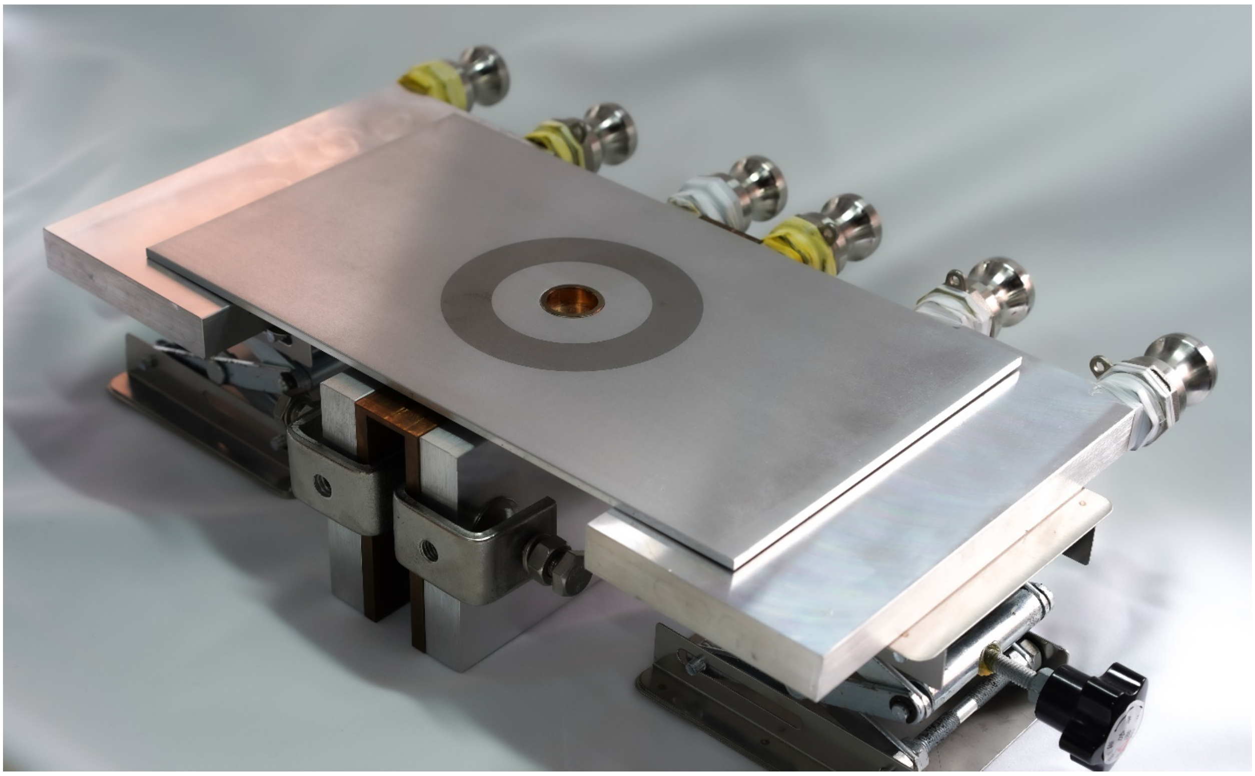

Supplementary Figure 4. Photograph of the experimental setup.
